# Cefepime induced acute interstitial nephritis – a case report

**DOI:** 10.1186/s12882-015-0004-x

**Published:** 2015-02-11

**Authors:** Kathy Mac, Ruchir Chavada, Sharon Paull, Kenneth Howlin, Jeffrey Wong

**Affiliations:** Department of Renal Medicine, South Western Sydney Local Health District, Liverpool, NSW 2170 Australia; Department of Microbiology and Infectious Diseases, South West Sydney Pathology Services, Liverpool Hospital, Liverpool, NSW 2170 Australia; Department of Medicine, Bankstown Hospital, Bankstown, NSW 2200 Australia

**Keywords:** Cefepime, Nephrotoxicity, Acute interstitial nephritis, Acute kidney injury

## Abstract

**Background:**

Nephrotoxicity due to drugs especially beta lactams and cephalosporins has been well recognised. Cefepime is a fourth-generation cephalosporin that is widely prescribed with few severe adverse reactions reported. Although cefepime induced neurotoxicity has frequently been reported, there is yet no reported case of acute interstitial nephritis caused by this drug. We report a biopsy proven case of acute kidney injury due to acute interstitial nephritis associated with use of cefepime for treatment of mastoiditis and temporal bone osteomyelitis.

**Case presentation:**

A 62-year-old Caucasian female presented with symptoms of right sided mastoiditis. A brain Magnetic Resonance Imaging scan revealed presence of right sided mastoiditis with concurrent temporal bone osteomyelitis. Microbiological specimen isolated an *Escherichia coli*. Therapy was commenced with intravenous cefepime. After 4 weeks of therapy with intravenous cefepime she developed acute kidney injury. No other medications were taken by the patient. Urine analysis revealed non-nephrotic range proteinuria. There was no red cell casts or white cell casts. Renal biopsy confirmed acute interstitial nephritis as cause of acute kidney injury. Cefepime therapy was ceased and treatment with ciprofloxacin was given to complete the treatment course. Renal function improved only partially with conservative management without any corticosteroid use. To our knowledge this is the first report of cefepime induced interstitial nephritis.

**Conclusions:**

Although cefepime has been considered to be a safe antibiotic from nephrotoxicity point, like other cephalosporins this adverse effect can occur rarely. Physicians need to be mindful of nephrotoxicity associated with its use and careful monitoring of renal parameters should be performed in patients on prolonged therapy with cefepime.

## Background

Acute kidney injury (AKI) from acute tubular necrosis and acute interstitial nephritis (AIN) are commonly seen in clinical practice [1]. Approximately 20% of AKI cases are caused by drug or toxins with potential increase in morbidity and permanent renal dysfunction [2]. Nephrotoxicity due to drugs can occur by various mechanisms including changes in glomerular hemodynamics, tubular toxicity, crystal nephropathy, obstruction, thrombotic microangiopathy and acute interstitial nephritis [3]. The majority of cases of AIN (75%) are caused by drugs with antibiotics accounting for approximately 25% of these [4].

Cefepime is a parenteral fourth-generation cephalosporin with an extended spectrum of antimicrobial activity that is routinely prescribed for variety of hospital acquired infections. It is considered well tolerated and is associated with few adverse reactions. Cefepime-related neurological toxicity is well described in patients with renal impairment [5]. However with the exception of a rat model with nephrotoxicity, till date no human report of nephrotoxicity exists [6]. PubMed search conducted with keywords- “cefepime”, “nephrotoxicity”, “acute interstitial nephritis”, “acute kidney injury” did not reveal any case reports in literature. We believe this is the first report of such adverse event arising from its use.

## Case presentation

**Figure 1:**
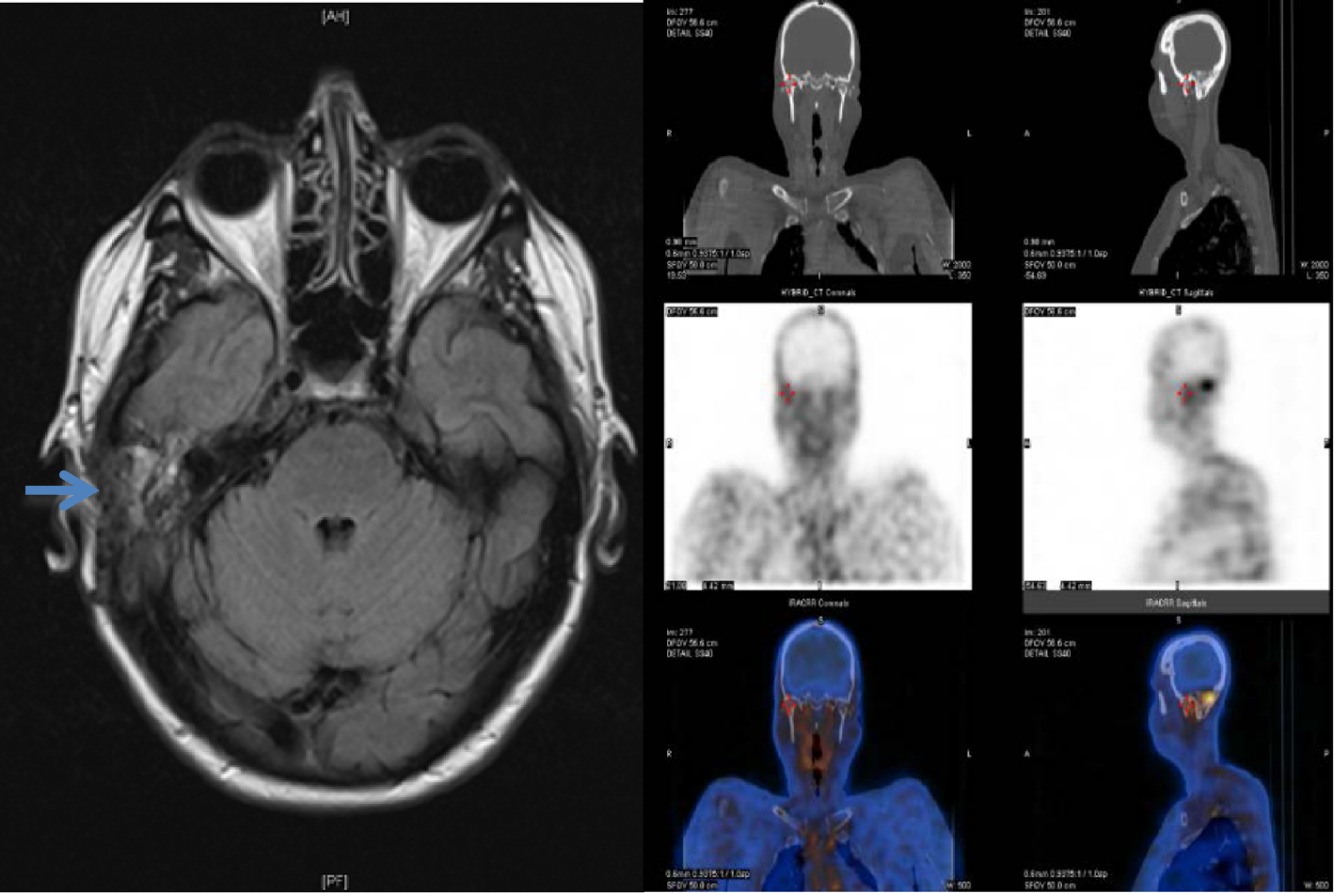
**MRI scan - T2 weighted image showing soft tissue oedema in the region of the right mastoid (left-see arrow) and technetium 99 m scan showing increased uptake in the same area (right).**

A 62 year old Caucasian female patient (height - 1.60 m, weight 72 kg, BMI 28.1) was admitted to hospital with AKI (patient’s baseline serum creatinine was 85 μmol/L and eGFR was 63 ml/min/1.73 m^2^ by MDRD equation) whilst on treatment for mastoiditis. Her background medical history included hypertension, and type 2 diabetes for which she was on regular sitagliptin-metformin 50/1000 mg half tablet twice a day, metoprolol 25 mg twice daily, lisinopril 10 mg daily and simvastatin 40 mg daily. She had a long medical history of recurrent otitis media for which she required grommet insertion. She was managed as outpatient with topical and oral ciprofloxacin by her otolaryngologist. Trial of oral trimethoprim-sulphamethaxazole (Bactrim DS) was prescribed without much clinical improvement. Eight weeks prior to this admission, she had symptoms of earache, localised post auricular swelling and fever. A CT scan showed mastoiditis and a contiguous subperiosteal abscess. A brain Magnetic Resonance Imaging (MRI) scan and Technetium 99 m labelled bone scan which revealed osteomyelitis (OM) of the petrous temporal bone (Figure 1). She underwent an emergency cortical mastoidectomy with drainage of the abscess and insertion of a new right ear grommet. Empirical therapy with ticarcillin-clavulanate (12.4 grams/day) was commenced.

A surface swab from the ear isolated *Escherichia coli* (which was sensitive to ticarcillin-clavulanate, piperacillin-tazobactam, cefepime and gentamicin, but resistant to ceftriaxone and ciprofloxacin). Susceptibilities were done using Vitek2 (Biomerieux, Marcy l’Etolie, France) using standard Gram negative card. Based on the antibiogram the isolate most likely had underlying extended spectrum beta lactamase, although confirmatory testing was not done as per current testing policy (non-sterile site isolate). The operative specimens of pus and tissue taken at the time of surgery remained sterile. Her antibiotic was changed to cefepime (6 grams/day).

**Figure 2:**
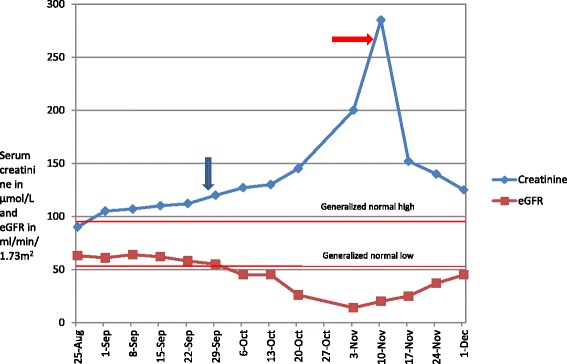
**Temporal trend in the creatinine and eGFR of our patient (from 1** ^**st**^ **admission till most recent follow up).** Note- the two lines ‘generalized high’ and ‘generalized low’ depict the values of serum creatinine. Blue arrow-Cefepime start date -28/9, red arrow Cefepime stop date -5/11.

She made good progress and was discharged with a plan of continuing cefepime for 6 weeks as treatment of OM on ambulatory care basis. After 2 weeks of therapy, she developed malaise and dysgeusia. At 4 weeks, she developed renal impairment (serum creatinine-140 μmol/L) and the cefepime dose was reduced to 4 grams/day. Her blood pressure at time was 130/82 mmHg. There was no fever, rash or peripheral oedema. Urine analysis done by automated iQ200 (Iris Diagnostics, Chatsworth, CA) machine and manual phase contrast microscopy showed WBC of <10 × 10^6^ /L, no RBC and no presence of either WBC/RBC casts. 24 hour urine collection (of 2.47 L) had proteinuria of 0.77 g/day (normal range-0.08-0.15 g/day). C reactive protein was 3 mg/L (normal < 5 mg/L). At week 5 into therapy, she was noted to have worsening AKI (serum creatinine 225 μmol/L) and was admitted for further investigation (Figure 2). She suffered the ‘injury stage’ of AKI according to Risk Injury Failure Loss of kidney function and End stage renal failure (RIFLE) criteria and stage 3 of Acute Kidney Injury Network (AKIN) classification at admission [7]. At this stage she was also noted to have serum potassium of 6.1 mmol/L (range 3.5-5 mmol/L) ,serum sodium of 148 mmol/L (range 135-145 mmol/L), serum chloride was 90 mmol/L (range 97-107 mmol/L) and blood urea of 15 mmol/L (range 2.5-7.5 mmol/L). There were no new medications including non-steroidal anti-inflammatory drugs (NSAIDs), chinese herbal supplements or any other form of naturotherapy that were taken by her.

**Figure 3:**
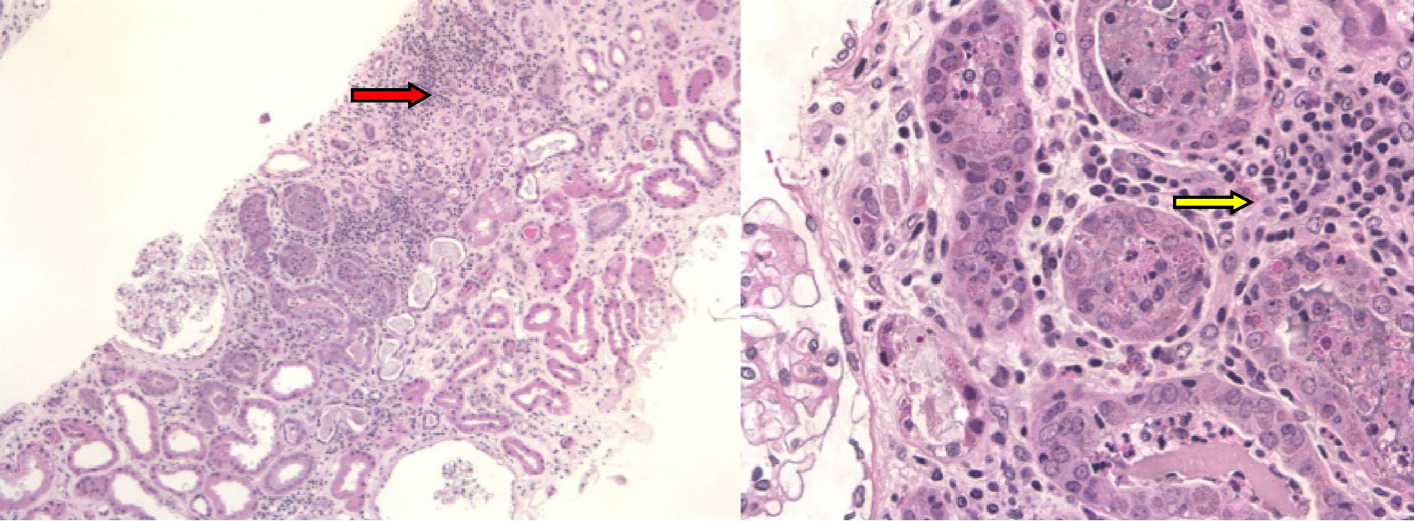
**Renal biopsy showed interstitial fibrosis with patchy areas of inflammatory cells infiltration.** Haematoxylin & Eosin stain x2.5, x40 hpf (red arrow-showing lymphocyte infiltration and interstitial fibrosis, yellow arrow-lymphocytes clusters).

During her inpatient stay all medications except metoprolol were ceased. Though she was clinically euvolaemic and suffered from non-oliguric renal failure, 36 hours trial of intravenous hydration with normal saline (0.9% w/v) at 80 ml/hour was administered with minimal effect on serum creatinine, blood urea and serum electrolytes. Marginal increase of urine output was noted which was commensurate with the intravenous fluid administered. Laboratory investigations of aetiology of AKI were negative or not detected. This included: Double stranded DNAs, ANCA, extractable nuclear antigens, rheumatoid factor, serum and urine protein electrophoresis, complement levels, immunoglobulin subclasses, streptococcal serology and hepatitis B and C serology. An ANA titre of 1:1280 (homogenous pattern) was noted and was thought to be related as a reaction due to cefepime therapy. Renal ultrasound revealed normal renal size, parenchyma and no obstructive uropathy. A renal biopsy showed moderate tubular atrophy with many intra-tubular casts in the medullary area. A few tubules were distended with cellular debris and acute inflammatory exudate (acute tubulitis). Moderate interstitial fibrosis was present with patchy areas of inflammatory infiltrates comprising predominantly lymphocytes, plasma cells and neutrophils. Eosinophils were not detected in the renal biopsy. Periodic acid Schiff stain and Gomori -silver stain were negative on the biopsy. Immunofluorescence staining was negative for IgA, IgG, IgM, kappa and lambda light chains, C1q, C3, amyloid and fibrinogen. Electron microscopy did not reveal any glomerular abnormality. (Figure 3)

It was concluded that this patient had drug-induced AIN, most likely related to cefepime. Cessation of the antibiotic led to a rapid improvement in her renal function over a week and therefore it was decided to treat her conservatively. The serum creatinine dropped from 295 μmol/L to 170 μmol/L within a week and then continued to trend downwards reaching up to 150 μmol/L on her 2 weekly follow-up post discharge. Her symptoms of malaise and dysgeusia had also abated at this visit. All the electrolytes (sodium, potassium and chloride) including blood urea were within normal ranges at this stage. Corticosteroid was not administered due to partially treated infection, diabetes and rapid improvement in renal function after cessation of antibiotics.

For completion of therapy (another 2 weeks) oral ciprofloxacin 500 mg twice daily was commenced, mindful of the fact that the isolate was resistant to ciprofloxacin with a minimal inhibitory concentration of 4 mg/L. Following discharge from hospital, she remained asymptomatic and continued to improve from her infection perspective. Sitagliptin-metformin, lisinopril and simvastatin were slowly reintroduced over the next 3 months after discharge while her renal function continued to improve.

Table 1 shows the temporal trend in the biochemical parameters including serum creatinine and eGFR. Although serum creatinine has plateaued, mild renal impairment still exists at 8 months of follow-up (serum creatinine of 130 μmol/L and eGFR of 56 ml/min/1.73 m^2^).

**Table 1:** **Changes in the biochemical parameters over course of time ND = Not done**

**Parameter**	**On admission to first hospital**	**On discharge from first hospital**	**During cefepime therapy**	**After cefepime cessation**
Serum Creatinine (μmol/L)	89	84	295	110
eGFR (mL/min/1.73 m^2^)	60	64	14	46
CRP (mg/L)	3.0	2.3	2.2	1.1
Blood Eosinophil count (10^^^9/L)	0.1	0.2	0.1	0.1
Spot urine protein (g/L)	ND	ND	0.55	0.3
Spot urine microalbumin/creatinine ratio (mg/mmol Cr)	ND	ND	45.5	ND
Spot urine protein/creatinine ratio (mg/mmol Cr)	ND	ND	103.8	45.2

This adverse drug reaction has been recorded permanently in her electronic medical records and also has been reported to Australian Therapeutic goods administration (TGA).

## Conclusions

We referred to the Naranjo criteria to report an adverse drug event [8]. A score of 6 was derived using this criterion rating this adverse reaction as “probable”. The limitation of these criteria to determine an adverse effect of an antibiotic is the difficulty of administering a placebo (when serious infection is present) and effects of dose reduction (serious consequences in sepsis). Nephrotoxicity was attributed to cefepime on basis on Naranjo criteria, clinical onset of AKI, absence of any other nephrotoxic drugs being administered and histopathological findings of the renal biopsy.

Cefepime nephrotoxicity has been described in dose-dependent manner in a rat model with increasing concentration of this antibiotic causing direct cytotoxic damage to the renal tubules, manifesting as proteinuria, glycosuria, and increase excretion of urinary sodium [6]. These could be attributed to the supratheurapeutic doses administered in the animal study. Except for proteinuria observed as altered urine microalbumin/creatinine ratio, other biochemical abnormalities were not observed in our patient. Cefepime was administered at maximally recommended dosage (of 6 grams/day) as for a ‘serious infection’ category which is also consistent with the product information sheet. Data for use of this antimicrobial in osteomyelitis is limited. Dosage regimens for 2 grams every 12 hourly have been used for osteomyelitis due to *Staphylococcus aureus* and 2 grams every 8 hours for Gram negative infections [9,10]. The development of AIN in our case could also be dose related as seen in the rat model as maximal doses of the antimicrobial were used to treat the patient. Although cefepime plasma levels of more than 22 mg/L have been found to be predictive of neurotoxicity, it’s correlation with therapeutic monitoring is far from clear as target trough concentrations have not been established and the stability of the drug itself in plasma is uncertain [11,12]. Optimal and validated methods for therapeutic drug monitoring of cefepime are needed before their routine use can be performed in clinical context.

Other animal model studies have attributed toxicity of cephalosporins to cortical AUC (area under the curve), direct effect of the lactam ring and disruption of the mitochondrial respiratory enzymes in the cortical tubular cells [13,14]. Different classes within cephalosporins have a different threshold of producing nephrotoxicity depending on their ionic charge and direct effect on the tubules [14,15]. Post marketing studies of cefepime raise the possibility of toxic nephropathy as a cephalosporin class effect; however this has not been described yet [16].

Most reports of antibiotic-related AIN describe its occurrence within the first three weeks particularly in the first ten days of commencing the drug. However, a well described delayed allergic reaction causing AIN has been reported with drugs such as proton pump inhibitors and mesalazine [17]. We hypothesize that two factors could have contributed to the delay in clinical presentation- 1) cefepime by itself acting as a hapten thereby eliciting delayed hypersensitivity reaction in the renal interstitium and 2) slow metabolism of cefepime which could have led to cumulative accumulation of N-methylpyrrolidine (NMP) (active metabolite) that is known to nephrotoxic in mice [18-20].

It is speculated that the lack of reported nephrotoxicity could be attributed to the protective mechanisms against oxidative damage (T suppressor cells) that exist in renal tubules [18]. However this could be mitigated in genetically predisposed individuals. Renal biopsies on cases of drug-induced AIN reveal a paucity of immune deposits, however presence of interstitial infiltrates containing lymphocytes suggest that cell mediated immunity (delayed hypersensitivity) plays an important role [20,21]. Interaction of tubular cells with lymphocytes increases interstitial inflammation. Both these phenomena were observed in the renal biopsy of our patient.

Although flank pain, haematuria and peripheral oedema have been described as common manifestation of AIN, they are present in <25% of cases [21,22]. None of these findings were observed in our patient who experienced malaise, dysgeusia early into her antibiotic therapy and subsequently went on to develop an AKI. Various prognostic factors have been studied in literature including severity of interstitial fibrosis, presence of granulomas and the fall of creatinine after AIN [23]. However they do not predict full recovery. Despite a rapid decline (over 2 weeks) in her serum creatinine after cessation of the antibiotic, our patient’s renal function did not recover to her baseline. Chronic impairment could be explained by interstitial fibrosis observed in her renal biopsy. Therapeutic role of corticosteroids is debatable with evidence for its use based on small, non-randomized case series. While some retrospective and uncontrolled studies have shown benefit of early administration of corticosteroids in preventing interstitial fibrosis, consistent benefits have not been observed in others [24,25]. We did not administer any adjunctive steroids as already mentioned.

In summary, this is the first human case report of cefepime induced AIN arising in the context of prolonged use of this antibiotic. Awareness of this adverse effect from this frequently prescribed antibiotic is important for the clinicians. We suggest careful monitoring of renal parameters in patients who are likely to receive a prolonged course of this antibiotic as signs and symptoms of AIN maybe subtle in the beginning of such an event.

## Consent

Written consent was obtained from the patient for this publication. A copy of written consent is available for review by the editor of this journal.

## Abbreviations

AIN: Acute interstitial nephritis
AKI: Acute kidney injury
